# Mucosa-Associated Microbiota in Gastric Cancer Tissues Compared With Non-cancer Tissues

**DOI:** 10.3389/fmicb.2019.01261

**Published:** 2019-06-05

**Authors:** Xiao-Hui Chen, Ang Wang, Ai-Ning Chu, Yue-Hua Gong, Yuan Yuan

**Affiliations:** ^1^Tumor Etiology and Screening Department of Cancer Institute and General Surgery, The First Hospital of China Medical University, Shenyang, China; ^2^Key Laboratory of Cancer Etiology and Prevention in Liaoning Education Department, The First Hospital of China Medical University, Shenyang, China; ^3^Key Laboratory of GI Cancer Etiology and Prevention in Liaoning Province, The First Hospital of China Medical University, Shenyang, China

**Keywords:** gastric cancer, microbiota, 16S rDNA, cancer microenvironment, risk

## Abstract

The link between microbiota and gastric cancer (GC) has attracted widespread attention. However, the phylogenetic profiles of niche-specific microbiota in the tumor microenvironment is still unclear. Here, mucosa-associated microorganisms from 62 pairs of matched GC tissues and adjacent non-cancerous tissues were characterized by 16S rRNA gene sequencing. Functional profiles of the microbiota were predicted using PICRUSt, and a co-occurrence network was constructed to analyze interactions among gastric microbiota. Results demonstrated that mucosa-associated microbiota from cancerous and non-cancerous tissues established micro-ecological systems that differed in composition, structure, interaction networks, and functions. Microbial richness and diversity were increased in cancerous tissues, with the co-occurrence network exhibiting greater complexity compared with that in non-cancerous tissue. The bacterial taxa enriched in the cancer samples were predominantly represented by oral bacteria (such as *Peptostreptococcus*, *Streptococcus*, and *Fusobacterium*), while lactic acid-producing bacteria (such as *Lactococcus lactis* and *Lactobacillus brevis*) were more abundant in adjacent non-tumor tissues. Colonization by *Helicobacter pylori*, which is a GC risk factor, also impacted the structure of the microbiota. Enhanced bacterial purine metabolism, carbohydrate metabolism and denitrification functions were predicted in the cancer associated microbial communities, which was consistent with the increased energy metabolism and concentration of nitrogen-containing compounds in the tumor microenvironment. Furthermore, the microbial co-occurrence networks in cancerous and non-cancerous tissues of GC patients were described for the first time. And differential taxa and functions between the two groups were identified. Changes in the abundance of certain bacterial taxa, especially oral microbiota, may play a role in the maintenance of the local microenvironment, which is associated with the development or progression of GC.

## Introduction

Gastric cancer (GC) is one of the most common malignant cancers and the third leading cause of cancer-associated death worldwide (Ferlay et al., 2015; Torre et al., 2015). The incidence of GC varies by region and race, with a high rate in East Asia. Both host factors (such as genetic susceptibility) and environmental factors (such as microbial infections) play crucial roles in gastric tumorigenesis (Compare et al., 2010). It is widely accepted that chronic *Helicobacter pylori* infection, which leads to enhanced inflammation and disorders of epithelial structure and function, is closely related to precancerous lesions such as atrophic gastritis. Nevertheless, only 1–3% of *H. pylori*-infected patients develop GC, and GC could not be completely prevented by the eradication of *H. pylori* (Wroblewski et al., 2010). Additionally, during the progression of GC, *H. pylori* colonization gradually decreases and even disappears (El-Omar et al., 1997). Therefore, biological factors other than *H. pylori* colonization may play an important role in the development of cancer and the maintenance of the local lesion microenvironment.

In recent years, the development of culture-independent technologies for characterizing microbiota composition has shed light on the profile of gastric microbiota. Studies have demonstrated the significant role played by non-*H. pylori* microbiota in gastric carcinogenesis in mice (Lofgren et al., 2011; Lertpiriyapong et al., 2014). In human studies, chronic *H. pylori* infection or the use of drugs such as omeprazole resulted in an elevated intragastric pH level by reducing the secretion of gastric acid, which allowed the proliferation and colonization of other bacteria (Mowat et al., 2000; Plottel and Blaser, 2011). As a result, the microbial balance of the gastric mucosa ecological niche was disrupted, and increased nitrosating species raised nitrite and *N*-nitrosamine levels in the stomach (Leach et al., 1987). Together, these findings highlighted the potential role of microbiota other than *H. pylori* in the development of GC.

Thus far, our understanding of the complex gastric flora in human is still limited. A few studies have revealed differences in the composition and function of the gastric microbiota between GC patients and control groups. However, there is no consensus on specific microbial taxa that play important roles in the pathogenesis of GC. In addition, microbial changes in the tumor microenvironment remain unclear. Unlike most previous studies that compared two groups of individuals, our research focused on the microbiota in the tumor microenvironment by comparing matched samples from GC patients. In this condition, the influence of the external environment and the genetic effects of the host would be controlled to some extent. We characterized the variations in the composition, interaction network and functions of gastric microbiota in cancerous and patient-matched non-cancerous tissues, aiming to explore the differential distribution profile of microbiota in the tumor microenvironment. Our findings will help to explore the role of mucosa-associated microbiota in carcinogenesis and in the maintenance of the local microenvironment in GC patients.

## Materials and Methods

### Study Population and Specimen Collection

A total of 124 gastric tissue samples, consisting of cancerous and paired non-cancerous tissues, were obtained from 62 GC patients who underwent subtotal gastrectomy at The First Hospital of China Medical University between 2012 and 2014. Patients who had received medical treatment (including probiotics, proton pump inhibitors, antibiotics, and H_2_ receptor antagonists) within 1 month, or those who had received chemotherapy or radiotherapy prior to the surgery were excluded. Gastric mucosa tissues, collected from the cancerous lesions and neighboring noncancerous sites (at least 5 cm away from the tumor site), were immediately frozen after surgical resections and stored at -80°C until further use. Epidemiologic information was obtained through questionnaire.

### DNA Extraction, PCR Amplification, and 16S rRNA Gene Sequencing

Total DNA was extracted using methods as previously described (Chen et al., 2017). After treating the mucosal samples with lysozyme, proteinase K, and SDS, we purified the DNA through multiple steps with phenol-chloroform-isoamylalcohol, then precipitated the DNA with glycogen, sodium acetate, and cold isopropanol, followed by cleaning the DNA with 70% ethanol. Finally, the DNA was dissolved in TE buffer and stored at -20°C. The V4–V5 regions of the 16S rRNA gene were amplified by primers 515F, 5′-barcode-GTGCCAGCMGCCGCGGTAA-3′ and 907R, 5′-barcode CCGTCAATTCMTTTRAGTTT-3′ (Liu et al., 2018), using the PCR kit (TransGenAP221-02, Peking; containing the high fidelity enzyme). PCR was performed as follows: 95°C for 5 min, followed by 34 cycles of 94°C for 60 s, 57°C for 45 s, and 72°C for 60 s, with final extension at 72°C for 10 min. In order to avoid possible contamination, DNA extraction and PCR set up were performed in a laminar air flow bench, illuminated with a UV lamp before use. Two negative controls (containing DNA extraction reagents and PCR kit reagents) were amplified and sequenced to assess contamination. The concentration and length of the PCR amplicons were detected by 2% agarose gel electrophoresis. PCR products with bright main strip (approximately 400–450 bp) were chosen for further experiments. The amplicons in the target region were purified with Qiagen Gel Extraction Kit (Qiagen, Germany). Sequencing libraries were generated by using DNA PCR-Free Sample Preparation Kit (Illumina, San Diego, CA, United States) following manufacturers recommendations and index codes were added. The library quality was assessed on the Qubit@2.0 Fluorometer (Thermo Scientific) and Agilent Bioanalyzer 2100 system. The libraries were sequenced on the Illumina Hiseq 2500 platform and 250 bp paired-end reads were generated. The sequence data have been deposited in the NCBI Sequence Read Archive (SRA) database with the accession number PRJNA532731.

### Processing of Sequencing Results and Taxonomical Annotation

The sequencing data were processed using the Quantitative Insights Into Microbial Ecology (QIIME, V1.9.1) pipeline as previously reported (Caporaso et al., 2010). Raw sequencing reads were assigned to each sample based on the unique barcode and identified as valid sequences. The low quality and short sequences were filtered out with the following criteria (Gill et al., 2006; Fadrosh et al., 2014): sequence reads with average Phred score ≤19, length less than 150 bp; paired reads having at least one with length less than 75% of their original length; reads with ambiguous bases; reads containing mononucleotide repeats more than 8 bp. Paired-end reads were assembled using FLASH (version 1.2.7) (Magoč and Salzberg, 2011). Chimeras were filtered out using UCHIME (v4.2.40) (Edgar et al., 2011). Sequence clustering analysis was performed using UPARSE pipeline (Edgar, 2013). Tags with at least 97% identity were clustered into the same operational classification unit (OTU; Supplementary Table S1). The Silva Database was used to annotate taxonomic information for OTU representative sequences by the ribosome database project (RDP) Classifier v.2.2.

### Microbial Diversity Analysis and Network Construction

QIIME (V1.9.1) was used to calculate diversity parameters. Alpha diversity analysis was performed to describe the richness and diversity of the microbiota in each sample. The Chao1 and ACE indices were used to estimate community richness, and the Shannon and phylogenetic diversity (PD) whole tree indices were applied to measure microbial diversity. Good’s coverage was used to evaluate the coverage quality of sequencing results. Beta diversity was measured by weighted UniFrac distance matrices and Bray–Curtis, and visualized via principal coordinate analysis (PCoA) and non-metric multidimensional scaling (NMDS) plots. Co-occurrence networks were structured by Spearman’s correlation analysis and visualized using the Cytoscape software (V.3.0.2., United States).

### Functional Prediction of Mucosal-Associated Microbiota

Functions of mucosal-associated microbiota were predicted using PICRUSt (Langille et al., 2013). Accuracy of the predicted metagenomes was evaluated by the nearest sequenced taxon index (NSTI; Langille et al., 2013). The enrichment analysis of pathways was performed based on Kyoto Encyclopedia of Genes and Genomes (KEGG) database. Additionally, predicted functional genes were also categorized into clusters of orthologous groups (COG), and compared across cancer and non-cancer groups by STAMP (Parks et al., 2014) to identify gene functions that differentiated bacterial communities in the two-group comparison.

### Statistical Analysis

Continuous variables that were not normally distributed were represented by inter-quartile range (IQR). Mann–Whitney *U* test was used to examine the correlation between alpha diversity parameters and epidemiological risk factors of GC. Tests were performed with SPSS 22.0 software (SPSS Inc., Chicago, IL, United States). Analysis of similarity (ANOSIM) and permutational multivariate analysis of variance (PERMANOVA) were performed to test the dissimilarity of beta diversity between groups by using ANOSIM and Adonis functions of vegan package in R (version 3.4.1). Linear discriminant analysis effect size (LEfSe) algorithm (Segata et al., 2011) was used to identify specific microbial taxa and functions that differed significantly between groups. Differences with linear discriminant analysis (LDA) scores >2.0 were considered significant. The analysis of differences in the abundance of the microbiota between two groups were also performed using the DESeq. 2 package in R (Weiss et al., 2017). The White’s non-parametric *t*-test was applied to determine statistical differences of COG between groups by STAMP. *P*-values were adjusted by Benjamini-Hochberg false discovery rate correction for multiple comparisons. *P* < 0.05 was considered statistically significant.

## Results

### Sequencing Results and Basic Characteristics of the Study Subjects

After PCR amplification, no bands were observed in the negative controls on the gel. The negative controls both had <130 reads, and the sequences could not be assembled. After sequencing and quality control, libraries of 16S rRNA gene V4–V5 region amplicon sequences from 61 cancerous and 62 adjacent non-cancerous tissue samples were used for further analysis, with an average of 67,958 effective tags per sample. The number of raw reads and effective tags for each sample are shown in Supplementary Table S2. At the 3% dissimilarity level, the number of OTUs were 152 (119-200) [median (IQR)] for the non-cancer group and 221 (177-350) for the cancer group. Good’s coverage was estimated to ensure quality assessment. All samples had a value >0.99, suggesting that the sequencing results were sufficient to represent the bacterial diversity of the bacteria in the gastric mucosa.

Detailed information regarding individuals included in the study is provided in Table 1. All cases were diagnosed as gastric adenocarcinoma. The median age of the patients was 60 years old. Samples with relative abundance of *H. pylori* more than 1% were identified as *H. pylori* sequencing positive, while others with relative abundance less than 1% were identified as *H. pylori* sequencing negative as previously proposed (Kim et al., 2015). Among the patients, *H. pylori* sequencing-positive cases accounted for 29%.

**Table 1:** Baseline characteristics of the study subjects (*n* = 62).

Characteristics	Median (IQR)/number (%)
Age (years)	60 (52–68)
<60	26 (42%)
≥60	36 (58%)
**Gender**	
Male	46 (74%)
Female	16 (26%)
**Family history**	
Yes	20 (32%)
No	42 (68%)
**Drinking**	
Nondrinker	38 (61%)
Drinker	24 (39%)
**Smoking**	
Never smoker	32 (52%)
Ever smoker	30 (48%)
***H. pylori* colonization status**	
Sequencing positive	18 (30%)
Sequencing negative	44 (70%)


*IQR, inter-quartile range.*

### Characteristics of Mucosa-Associated Microbiota in Cancerous Tissues

#### Microbial Community Profile of Cancerous Tissues

*Proteobacteria* was the predominant phylum in the cancerous samples, followed by *Firmicutes*, *Bacteroidetes*, *Actinobacteria*, *Acidobacteria*, and *Fusobacteria* (Table 2). Overall, 90% of cancerous samples were dominated by *Proteobacteria*, with relative abundance >50% in each case. Four samples were dominated by *Firmicutes* or *Bacteroidetes*, while the remaining two samples had no obviously dominant phylum (Supplementary Figure S1).

**Table 2:** The relative abundances of major bacterial phyla in cancerous and adjacent non-cancerous tissues.

Taxonomy	Non-cancer group (%)	Cancer group (%)	*P*
*Proteobacteria*	83.691	78.434	0.084
*Firmicutes*	1.907	5.568	0.000
*Bacteroidetes*	0.518	2.339	0.000
*Actinobacteria*	0.080	0.741	0.000
*Fusobacteria*	0.041	0.257	0.000
*Acidobacteria*	0.004	0.314	0.000


*The P-value is calculated using Wilcoxon signed-ranks test.*

#### Correlation Between Cancerous Tissue Microbiota and GC Risk Factors

We next analyzed the association between GC-related epidemiological risk factors (such as age, gender, smoking, alcohol consumption, family history, and *H. pylori* colonization status) and the microbiota. Results showed that in tumor tissues, the alpha diversity of the microbiota, estimated by Shannon index and PD whole tree, was significantly increased in GC patients aged over 60 years old compared with that of younger patients (*P* = 0.043, 0.022, respectively, Supplementary Table S3). No significant differences in the microbial richness or community structure were discovered in relation to the other risk factors.

#### Ecological Network of Gastric Microbiota in Cancerous Tissues

**FIGURE 1:**
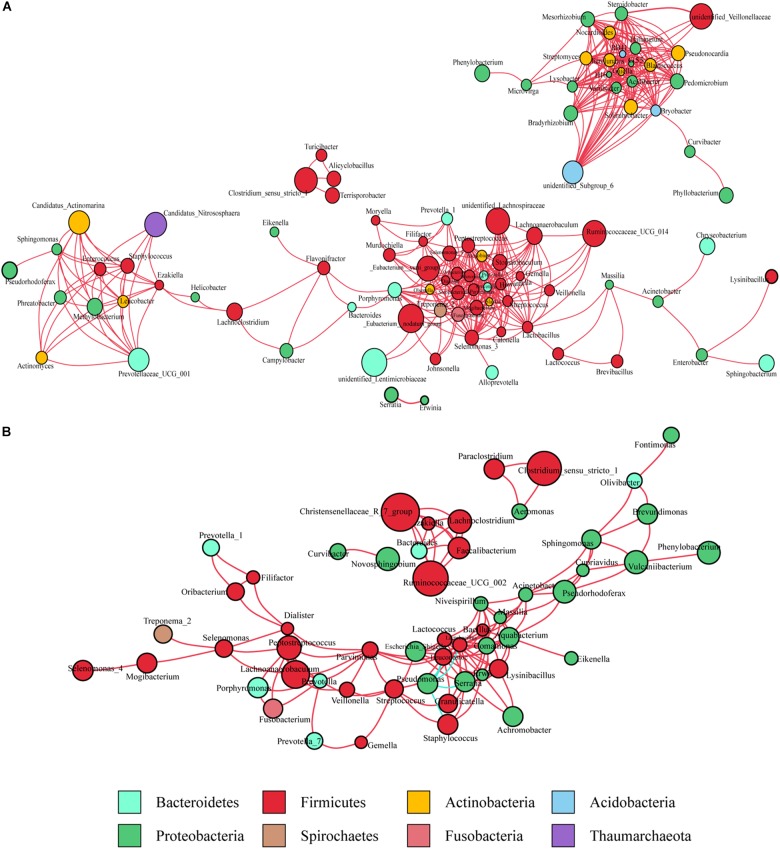
Co-occurrence network analysis of gastric bacterial genera with correlation coefficient >0.6 or < –0.6 **(A)** in cancer tissues and **(B)** non-cancer tissues. The nodes represent different genera, whose colors indicates different phyla. The size of node shows relative abundance of the genus. Positive and negative correlations are drawn in red and blue, respectively.

Co-occurrence network analysis was used to describe the interactions among the microbiota in the complex gastric microbial population. As shown in Figure 1A, the interactions across the mucosa-associated microbiota mainly occurred among taxa belonging to the phyla *Firmicutes* and *Proteobacteria*, the two predominant phyla in the taxonomic profiles. Collectively, co-occurrence interactions dominated in the networks. In addition, co-occurrence interactions between *Helicobacter* and *Lachnoclostridium* and between *Helicobacter* and *Ezakiella* were also observed in the cancer tissue network. No co-exclusion interaction was identified in strong correlations (*r* > 0.6 or <-0.6).

### Characteristics of Mucosa-Associated Microbiota in Non-cancerous Tissues

#### Microbial Community Profile of Non-cancerous Tissues

The gastric microbiota in non-cancerous tissues was also dominated by *Proteobacteria* and consisted mainly of the six main phyla observed in the cancer group. However, the ranking of phyla differed slightly (Table 2), with a decreased abundance of *Firmicutes*, *Bacteroidetes*, *Actinobacteria*, *Acidobacteria*, *Fusobacteria* (all *P* < 0.01), but an increased abundance of *Proteobacteria* (*P* = 0.084) when compared with the cancer group.

#### Correlation Between Non-cancerous Tissue Microbiota and GC Risk Factors

Unlike the cancer group, *H. pylori* colonization significantly impacted the composition of the microbiota in the non-cancerous samples. The *H. pylori* sequencing-positive group showed greater bacterial diversity (Shannon index) than the sequencing-negative group (*P* = 0.022, Supplementary Table S4). The differences in microbial structure were assessed by Bray–Curtis and weighted UniFrac distance matrices. PERMANOVA showed significant differences between *H. pylori* sequencing positive and negative group (Bray–Curtis, *P* = 0.001; weighted UniFrac distance matrices, *P* = 0.004). No significant association was found between microbial richness or community structure and the other risk factors.

LEfSe analysis (Segata et al., 2011) was conducted to further explore the taxa correlated with *H. pylori* colonization status in non-tumor tissues (Supplementary Figure S2). In the *H. pylori* sequencing-positive group, the enrichment of 15 genera was observed other than *Helicobacter*. Among them, *Serratia*, *Lactobacillus*, and *Streptococcus* were abundant in all non-cancerous tissues, ranking in the top 20 abundant genera. In comparison, *Pseudomonas aeruginosa* and its higher level taxa from genus to phylum were significantly more abundant in the *H. pylori* negative group. In summary, *H. pylori* colonization status, which is a well-known epidemiological risk factor for GC, was closely associated with the composition and structure of the gastric microbiota.

#### Ecological Network of Gastric Microbiota in Non-cancerous Tissues

As observed in the cancerous tissue samples, the interactions within the microbiota of the non-cancerous samples also occurred mainly in the two taxonomically dominant phyla, *Firmicutes* and *Proteobacteria*; however, fewer phyla were involved. The network diagram revealed denser and more complicated co-occurrence interactions across the microbiota in the cancerous tissues compared with the non-cancerous tissues, especially with regard to oral bacteria (such as *Streptococcus*, *Peptostreptococcus*, *Fusobacterium*, *Dialister*, and *Prevotella*) (Figure 1A,B). However, several co-exclusion interactions were observed in the non-cancerous tissue network, with *Pseudomonas* as the interaction node. These interactions occurred among *Pseudomonas and Serratia*, *Lactobacillus*, *Lactococcus*, *Staphylococcus*, as well as *Leuconostoc.*

### Differential Microbial Taxa of the GC Tissues Compared With the Non-cancerous Tissues

#### Bacterial Taxonomic Richness and Diversity

**FIGURE 2:**
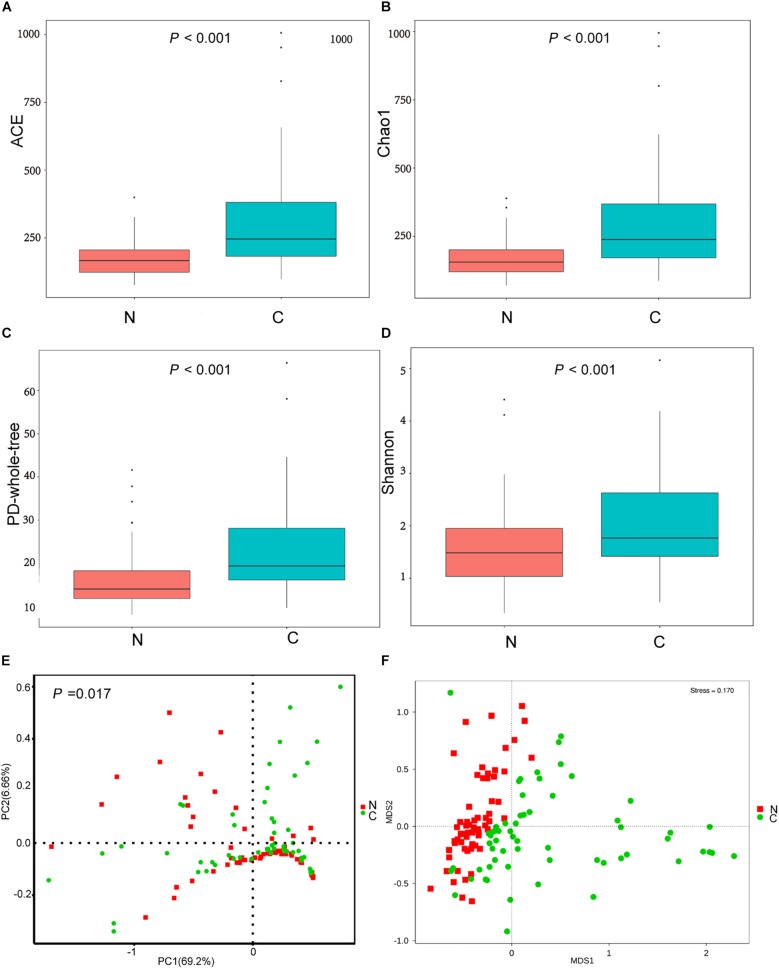
**(A–D)** The alpha diversity of the microbial communities in cancer and non-cancer groups. The global microbial structure differs between the two groups in **(E)** PCoA plot and **(F)** NMDS plot. C, cancer group; N, non-cancer group.

The cancerous tissues had a significantly higher number of OTUs than the non-cancer tissues (219 versus 148 OTUs; *P* < 0.05). In terms of alpha diversity (Figure 2A–D), compared with non-cancerous tissues, cancer samples had significantly increased community richness, which was estimated by Chao1 and ACE index (both *P* < 0.001), and diversity, which was estimated by Shannon index and PD whole tree (both *P* < 0.001).

#### Bacterial Community Structure

To analyze differences in microbial community structure between groups, we assessed the beta diversity (Figure 2E,F). The overall differences were visualized using PCoA and NMDS plots. The diversity described in the PCoA plots by the top two principal coordinates was 75.86% based on weighted UniFrac phylogenetic distance matrices. The non-cancerous and cancerous samples were clustered separately, with a significant difference confirmed by ANOSIM (*P* = 0.017, Figure 2E). The results of NMDS analysis based on OTU level also divided samples into two separate clusters (Figure 2F), suggesting significant differences in the overall community structure of mucosal microorganisms between the cancer and non-cancer groups.

#### Specific Microbial Taxa Associated With GC in the Cancer Microenvironment

**FIGURE 3:**
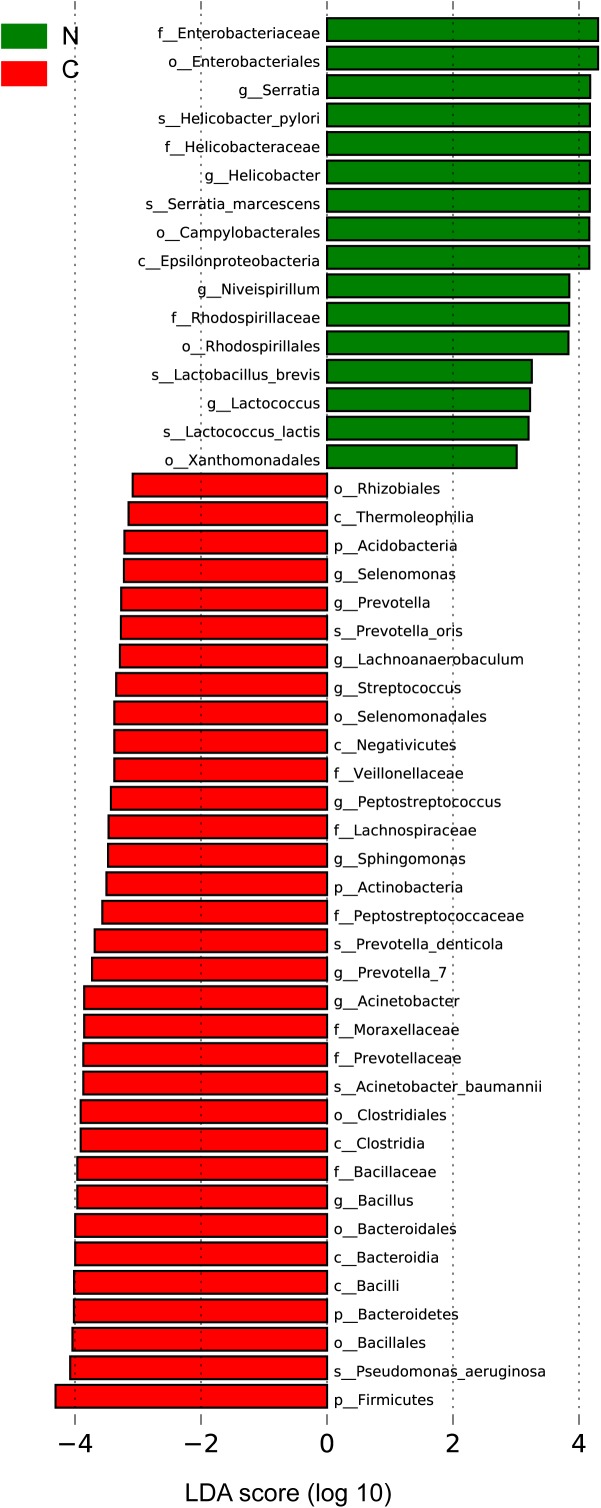
Differential bacteria between the two groups by LEfSe analysis (LDA scores >3.0). Green indicates taxa enriched in non-cancerous tissues and red indicates taxa enriched in cancerous tissues. C, cancer group; N, non-cancer group.

We sought to identify the differential microbiota between the two sample groups, using two different methods. First, the LEfSe analysis (Segata et al., 2011) was conducted to identify the specific taxa responsible for the statistically significant differences (Figure 3). Overall, 49 taxa were identified as being differentially abundant between the cancer and non-cancer samples at the phylum, class, order, family, genus, and species levels (LDA = 3). 33 of them were enriched in the cancer group, including the genera *Streptococcus*, *Peptostreptococcus*, *Prevotella*, *Prevotella_7*, *Acinetobacter*, *Bacillus*, *Selenomonas*, *Lachnoanaerobaculum*, and *Sphingomonas* and the species *Acinetobacter baumannii*, *P. aeruginosa*, *Prevotella oris*, and *Prevotella denticola*, most of which were oral microbiota. 16 taxa were enriched in the non-cancer group, including the genera *Serratia*, *Helicobacter*, *Niveispirillum*, and *Lactococcus*, and the species *H. pylori*, *Serratia marcescens*, *Lactococcus lactis*, and *Lactobacillus brevis*.

Then, we used the DESeq. 2 package to calculate and compare the top 20 abundant genera with a median relative abundance >0.1% between the two groups. In addition to the eight genera (*Peptostreptococcus*, *Streptococcus*, *Acinetobacter*, *Bacillus*, *Bacteroides*, *Sphingomonas*, *Prevotella_1*, and *Prevotella_7*) described above as being enriched in the cancer group, *Fusobacterium* was also shown to be significantly more abundant in cancerous tissues. On the other hand, *Helicobacter* and *Lactobacillus* showed significant increase in the adjacent non-cancerous tissues (Supplementary Table S5).

**FIGURE 4:**
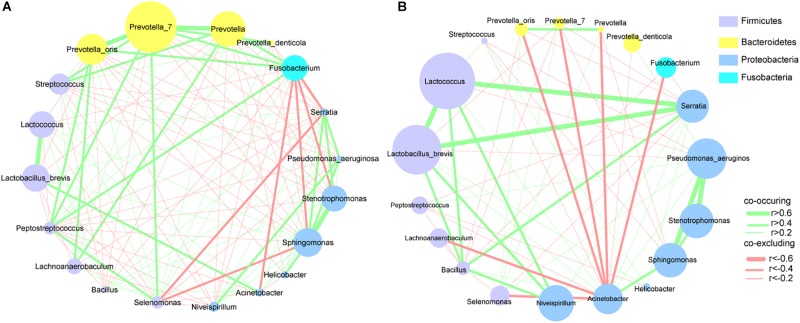
Correlation networks of differential bacteria **(A)** in cancer and **(B)** non-cancer tissues. A subset of significant correlations with strengths of at least 0.2 were selected for visualization. The size of the nodes correspond to weighted node connectivity (WNC) scores.

To explore the interactions among these differential bacteria, the network within each group was constructed (Figure 4A,B). We found that co-occurrence and co-excluding interactions were significantly different between the two groups. Oral microbiota including *Prevotella*, *Prevotella_7*, *Peptostreptococcus*, *Streptococcus*, and *Fusobacterium* had higher weighted node connectivity (WNC) scores in cancerous tissues (Figure 4A), while *Serratia*, *Lactococcus*, and *L. brevis*, which were identified above as being enriched in non-cancer group, had higher WNC scores in non-cancer tissues (Figure 4B). Higher WNC scores indicated centralities and their important roles in the interaction network.

### Functional Prediction of Mucosal-Associated Microbiota

Based on 16S rRNA gene sequencing data, PICRUSt was performed to predict the functional profiling of microbial communities using the KEGG databases (Langille et al., 2013). The NSTI scores (0.02–0.13) (Langille et al., 2013) demonstrated a reasonable prediction accuracy (Supplementary Table S6). The LEfSe algorithm was used to detect differences in the functional pathways of the microbiota between the cancer and non-cancer groups. Several metabolic pathways were enriched in the cancerous samples, including those involved in nucleotide metabolism (pyrimidine and purine metabolism), energy metabolism (methane metabolism), carbohydrate metabolism (e.g., glycolysis and gluconeogenesis), etc. (Supplementary Figure S3), while other predicted pathways were significantly increased in the non-cancerous samples, such as bacterial motility (motility proteins and chemotaxis), membrane transport (e.g., secretion system and phosphotransferase system), lipopolysaccharide biosynthesis and signal transduction (two component system), etc. (Supplementary Figure S3).

Given that the accumulation of nitrogen-containing compounds such as nitrate and nitrite in the stomach can increase the risk of GC and promote the malignant transformation of cells in the stomach (Correa, 1992; Alarcon et al., 2017), we focused on the microbial functions relevant to the metabolism of nitrogen-containing compounds in the cancerous and non-cancerous tissues. Compared with the non-cancer group, metabolic enzymes related to denitrification, including nitrate reductase (COG1116) and nitrous oxide reductase (COG4263), were enriched in the gastric microbiota of the cancer group (Supplementary Table S7).

## Discussion

Microbial communities have been universally considered as an important biological factor in the occurrence and development of GC. Recent studies have shown the changes in the microbial populations of GC patients compared with control groups (Eun et al., 2014; Coker et al., 2018; Ferreira et al., 2018; Hsieh et al., 2018). However, microbial profiles in the tumor microenvironment were still unclear. Our findings confirmed that gastric mucosa-associated microbiota from cancerous and adjacent non-cancerous tissues established distinct micro-ecological systems. We observed significant microbial community disturbances in the cancer lesions, where the richness and diversity of the microbial communities increased significantly, the interaction network exhibited greater complexity, and the relative abundance of *H. pylori* decreased compared with non-cancerous tissues. The bacterial taxa enriched in the cancer group mostly consisted of oral bacteria (such as *Peptostreptococcus*, *Streptococcus*, and *Fusobacterium*), while lactic acid-producing bacteria (such as *L. lactis*, and *L. brevis*) were more abundant in the adjacent non-tumor tissues. These results suggest that the occurrence and development of GC disturbs the structure of the endogenous bacterial community, and that *H. pylori* might play a limited role in the development and/or progression of malignant tumors.

### Characteristics of Mucosal-Associated Microbiota in GC and Non-cancerous Tissues

Changes in localized niches in GC patients result from long-term interactions among microorganisms, the host, and the environment. Shifts in gastric acidity and nutrient availability, as well as the innate immune response all contribute to the disruption of the microbial ecological balance in GC patients, leading to the colonization and overgrowth of non-*H. pylori* bacteria (Brawner et al., 2014). Gastric microbiota in cancerous and adjacent non-cancerous tissues were both dominated by *Proteobacteria* at the phylum level. The significant differences between the two groups mainly occurred at the genus and species level. Oral bacteria such as *Fusobacterium*, *Streptococcus*, *Peptostreptococcus*, and *Prevotella* were enriched in cancerous tissues. On the other hand, *Serratia* and lactic acid producing bacteria such as *Lactococcus* and *Lactobacillus* were more abundant in non-cancer group. Importantly, we identified some differential taxa that have not been reported in gastric microbiota studies before, such as *P. denticola*, *P. aeruginosa*, and *Serratia*. Although *Streptococcus*, *Peptostreptococcus*, *Fusobacterium*, and *Lactobacillus* have been discussed in recent studies of GC (Castano-Rodriguez et al., 2017; Hsieh et al., 2018), our research is the first to identify their abundance differences in the tumor microenvironment.

Our results highlight the possible pathogenic role of oral microbiota in GC. The changed acidity environment of GC may provide increased opportunities for oral bacteria to colonize the gastrointestinal tract. Previous studies have shown that oral bacteria were associated with colorectal cancer (CRC; Nakatsu et al., 2015) and pancreatic cancer (Michaud, 2013), which attracted widespread attention. Patients with certain oral pathogens had a higher risk of developing pancreatic cancer (Ertz-Archambault et al., 2017). There has been no studies directly analyzing changes in the oral microbiota of GC patients. A more in-depth investigation is needed to characterize its role as a driver or passenger in carcinogenicity. Whether oral microbes could be used as a non-invasive diagnostic marker for GC requires further studies.

It is noteworthy that *Fusobacterium* was more abundant in GC specimens than non-cancerous tissues. *Fusobacterium* is a genus of anaerobic bacteria closely related to CRC. Increasing evidence has shown its roles in carcinogenesis, diagnosis, progression and prognosis of CRC (Zhang et al., 2018). A recent report revealed that *Fusobacterium* species were over-represented in GC patients, and that it could be used as a diagnostic marker for GC. It supported our findings, despite being based on a relatively small cohort (11 GC patients vs. 16 controls) (Hsieh et al., 2018).

Previous reports had shown the significant increase in the abundance of *Lactobacillus* species in GC compared with the control population (Aviles-Jimenez et al., 2014; Eun et al., 2014; Wang et al., 2016; Castano-Rodriguez et al., 2017). However, changes in lactic acid producing bacteria in the tumor microenvironment were previously unknown. We observed an obvious enrichment of lactic acid producing bacteria (such as *L. lactis* and *L. brevis*) in non-cancerous tissues compared with cancerous tissues. *Lactococcus* and *Lactobacillus* species are generally thought of as probiotics and considered beneficial to the host. Reports show that lactic acid production has immunomodulative, anti-cancer and anti-inflammatory activities, and is conducive to the eradication therapy of *H. pylori* (Kim et al., 2014; Han et al., 2015; Kanayama et al., 2018). Another noteworthy taxon enriched in the non-cancer tissues here was *S. marcescens*, which has not been reported in cancer-related microbiota research before. Prodigiosin, a secondary metabolite of *S. marcescens*, could induce GC cell apoptosis and inhibit human oral squamous carcinoma cell growth *in vitro* (Diaz-Ruiz et al., 2001; Cheng et al., 2017).

#### Correlation Between Microbial Community Characteristics and GC Risk Factors

Results of association analyses between epidemiological risk factors and gastric microbiota revealed an increased Shannon’s diversity in the older patients (≥60 years old). This may be due to the overall decline in immunity associated with age, and the elevated pH caused by local mucosal atrophy, both of which are conducive to bacterial growth (Sheh and Fox, 2013). We also showed that sex, smoking, drinking, and family history of upper gastrointestinal cancer were not significantly associated with microbial community characteristics. In addition, *H. pylori* colonization changed the microbial community structure, with a significant increase in alpha diversity, which was observed in *H. pylori* sequencing-positive group compared with negative group. This finding is supported by a previous report showing that the abundance of *H. pylori* can remarkably affect the diversity of the gastric microbiota (Wang et al., 2016). How *H. pylori* impacts on the diversity and structure of the gastric microbiota is not yet understood. Nevertheless, it is plausible that changes in the gastric niche induced by *H. pylori* may influence the colonization and growth of other microbes.

### Ecological Networks of Microbial Taxa in Cancer and Adjacent Mucosal Tissues

The microbiota inhabiting the mucosal surface affects the development of cancer by altering the metabolome and regulating cell proliferation and tumor growth (Johnson et al., 2015). A niche-specific microbial network could affect the disease-associated microenvironment. A study has shown stronger interactions among differential OTUs in the microbiota of GC patients compared with those in patients with precancerous lesions (superficial gastritis, atrophic gastritis, intestinal metaplasia) (Coker et al., 2018). We extended previous work by delineating the interaction networks of microbiota in cancerous and adjacent non-cancerous tissues. Our results revealed that the distribution of microbial interactions differed between cancerous and adjacent non-cancerous mucosae. In the strong correlation network (*r* > 0.6 or *r* < -0.6), compared with the non-cancer group, more microbial taxa were involved in the cancer group. And they formed a denser and more complex association network, in which only co-occurrence interactions were observed. This may be due to the decrease in abundance of *H. pylori*, the increased abundance of other microbes in cancerous tissues, and shifts of the local pathogenic microenvironment. The enrichment of a larger number of microbial taxa, particularly oral microbes, in the cancerous samples contributed to the formation of a disease-specific interaction network. Further, the markedly increased symbiotic interactions in the cancer group might also contribute to the maintenance of the tumor microenvironment and even affect further disease development. Interestingly, strong co-occurrence interactions formed by *Streptococcus*, *Peptostreptococcus*, *Fusobacterium*, *Dialister*, and *Prevotella*, showed the centralities of these taxa in the whole cancer group network. They also played significant roles in the network constructed of the differentially abundant bacteria. These results suggest that these oral microbiota may have major impact on the structure of the microbiota in GC patients, which deserves further investigations. On the other hand, several co-exclusion interactions presented in the whole non-cancer group network, occurred separately between *Serratia*, *Lactobacillus*, *Lactococcus* with *Pseudomonas* (as the interaction node). Moreover, *Serratia*, *Lactobacillus*, and *Lactococcus* exhibited their centralities in the network constructed of the differentially abundant bacteria. These findings show their potential protective effects.

### Functional Analysis of Mucosal-Associated Microbiota in GC and Non-cancer Tissues

Our work showed differences in the predicted microbiota functions in cancerous and adjacent non-cancerous tissues. Purines are rich in the cancer microenvironment, with the capability of regulating immune cell responses and the release of cytokines (Di Virgilio, 2012). In this study, the purine metabolism pathways were enriched in the cancer group, indicating the metabolism of released purines in tumor microenvironment by GC microbiota (Coker et al., 2018). In addition, the microbiota in cancerous tissues had an increase in denitrification functions compared with non-cancerous tissues. The abundant nitrate reductase (COG1116) is associated with bacterial-mediated N-nitrosylation (Hillman, 2004), while the N-nitroso compound is a causative factor in carcinogenesis. Additionally, several pathways that facilitated host cell recognition were decreased in the microbiota of cancerous tissues, such as bacterial movement (bacterial motor proteins and chemotaxis) and bacterial signal transduction (membrane transport, etc.). To develop a deeper understanding of gastric carcinogenesis, further studies are needed to examine the significance of microbial functional variations in the GC microenvironment.

### Advantages and Limitations

In this study, gastric mucosa samples were obtained from GC patients undergoing surgical treatment, thus avoiding possible oral microbial contamination that may occur during upper digestive endoscopy sampling. Our work provided insights into the composition, function and interaction network of the mucosa-associated bacterial community in the tumor microenvironment, and its links with GC risk factors. We identified specific genera and species that may be involved in gastric carcinogenesis and the maintenance of the tumor microenvironment. However, this study had several limitations. Firstly, PICRUSt, which was used for microbial functional assessment, is a predictive method by nature. Although it has been widely applied in studies of disease-associated microorganisms, this approach may not fully reflect the biological functions of the microorganisms. Furthermore, this study did not include gastric tissues from individuals without GC for comparison. However, to a certain extent, this reduced the impact of inter-subject dissimilarity. In addition, our DNA extraction protocol did not include a bead-beating step, which was an extra cell lysis process to destroy the hard-to-break cell membranes of certain species. A previous study had compared DNA extraction methods with and without a bead-beating step. The result demonstrated that the extraction method without a bead-beating step inevitably missed some taxa with hard-to-break cell membranes, however, these taxa were exceedingly rare and would not have a detrimental impact on the final results (Yu et al., 2017). The research revealed the correlation between gastric microbiota and GC, but could not determine the causal relationship. This will require follow-up animal models and cell culture experiments.

## Conclusion

Compared with non-cancerous tissues, mucosa-associated microbiota in cancer tissues showed significant differences in distribution profile. The alterations in microbial community composition, function and ecological network in GC tissues may be involved in carcinogenesis and the maintenance of local microenvironment of GC. In future studies, we would focus on verification using a larger number of samples and multicentric populations, and extend our work into cell culture systems and animal models to examine the pathogenic roles of microorganisms in GC. These investigations into the mucosa-associated microbiota of GC patients may contribute to the development of new strategies for prevention, diagnosis, early intervention, and treatment of GC.

## Ethics Statement

This study was approved by the Human Ethics Review Committee of The First Hospital of China Medical University (Shenyang, China [2012]115), and written informed consent was obtained from all patients.

## Author Contributions

YY contributed conceptualization, funding acquisition, project administration, writing–review, and editing. X-HC contributed data curation, investigation, formal analysis, and writing the original draft. AW contributed methodology and software. A-NC partly contributed to the validation. Y-HG contributed resources and supervision.

## Conflict of Interest Statement

The authors declare that the research was conducted in the absence of any commercial or financial relationships that could be construed as a potential conflict of interest.

## Supplementary Material

FIGURE S1 Bacterial profiling plot of relative abundances of operational taxonomic units (OTUs) at the phylum level.

FIGURE S2 LEfSe analysis in non-cancer tissues with and without *H. pylori* colonization. Green indicates taxa enriched in *H. pylori* positive group and red indicates taxa enriched in *H. pylori* negative group.

FIGURE S3 Differences in functional compositions between the cancer and non-cancer groups by LEfSe analysis (LDA scores >2.0). Green indicates functions enriched in cancer group and red indicates functions enriched in non-cancer group. C, cancer group; N, non-cancer group.

TABLE S1 OUT abundance table.

TABLE S2 Sequencing data.

TABLE S3 Association between alpha diversity indices of microbiota in cancerous tissues and GC risk factors.

TABLE S4 Association between alpha diversity indices of microbiota in non-cancerous tissues and GC risk factors.

TABLE S5 Significantly differential bacteria identified among the top 20 abundant genera of the cancer and non-cancer groups.

TABLE S6 Nearest sequence taxon index (NSTI) scores for all samples.

TABLE S7 Comparison for microbial functions associated with nitrogen-containing compound metabolism between cancer and non-cancer groups.
