# Supplementary figures and images for: Mucosa-Associated Microbiota in Gastric Cancer Tissues Compared With Non-cancer Tissues
Source: Front Microbiol. 2019 Jun 5;10:1261. doi: 10.3389/fmicb.2019.01261 (PMC6560205; doi:10.3389/fmicb.2019.01261)

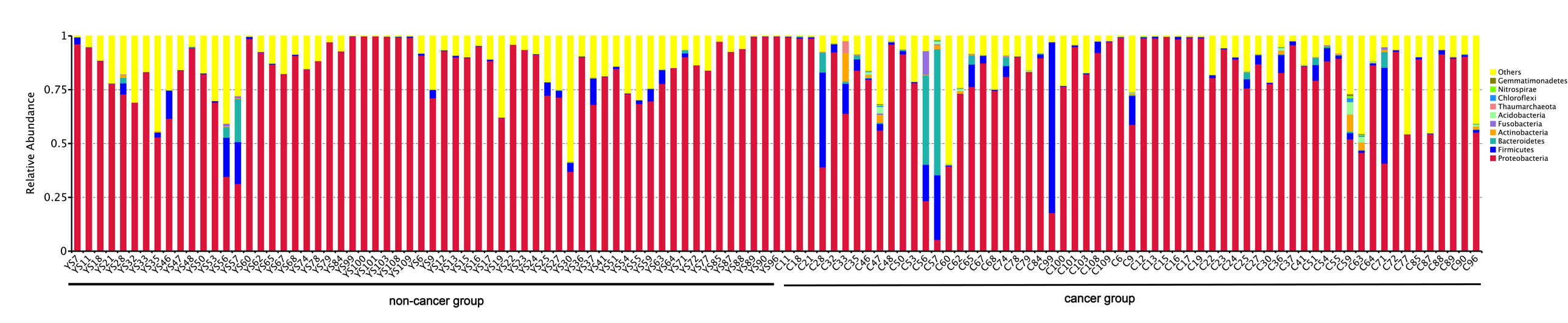

Supplement: FIGURE S1 — Bacterial profiling plot of relative abundances of operational taxonomic units (OTUs) at the phylum level. [file Image_1.JPEG]

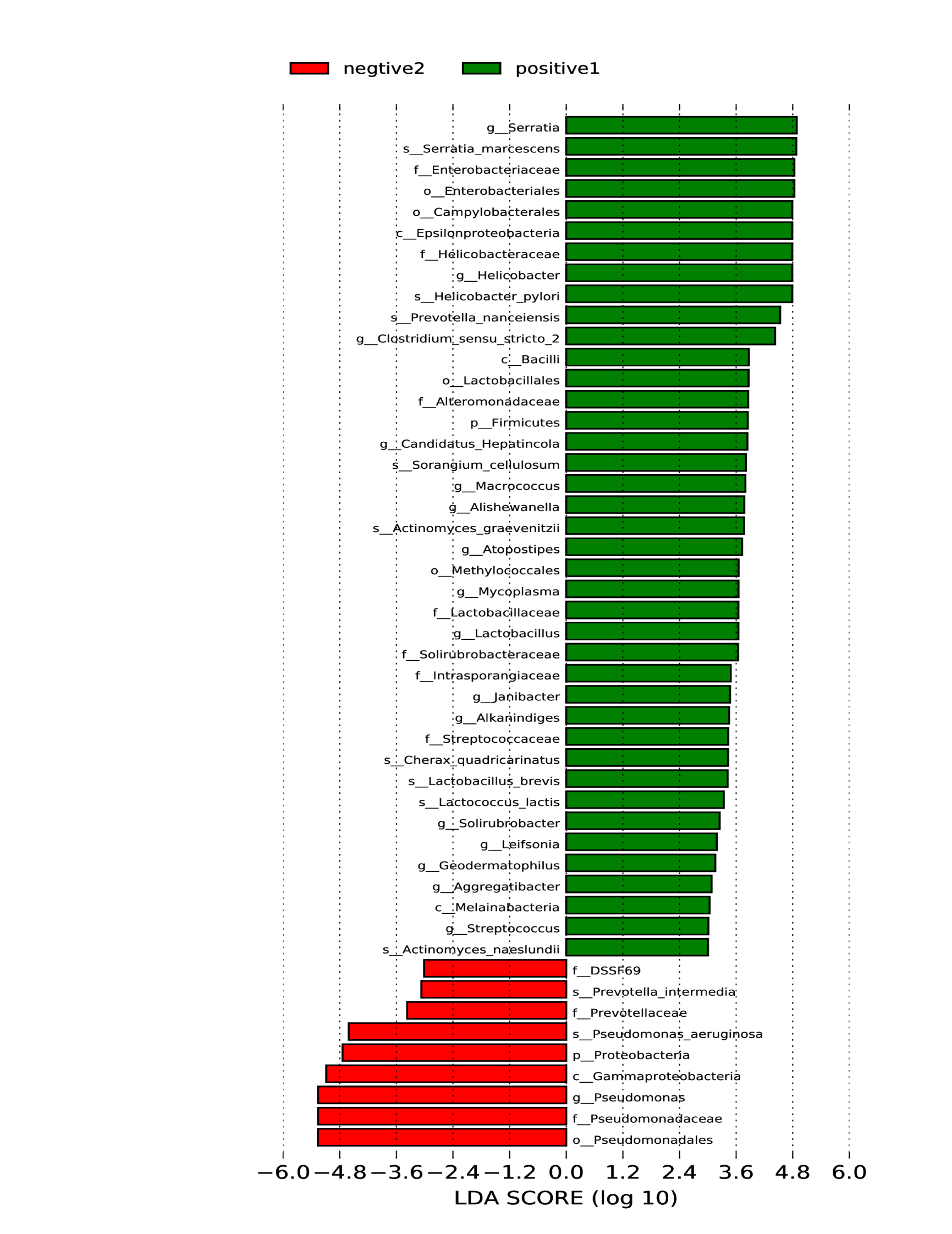

Supplement: FIGURE S2 — LEfSe analysis in non-cancer tissues with and without H. pylori colonization. Green indicates taxa enriched in H. pylori positive group and red indicates taxa enriched in H. pylori negative group. [file Image_2.TIF]

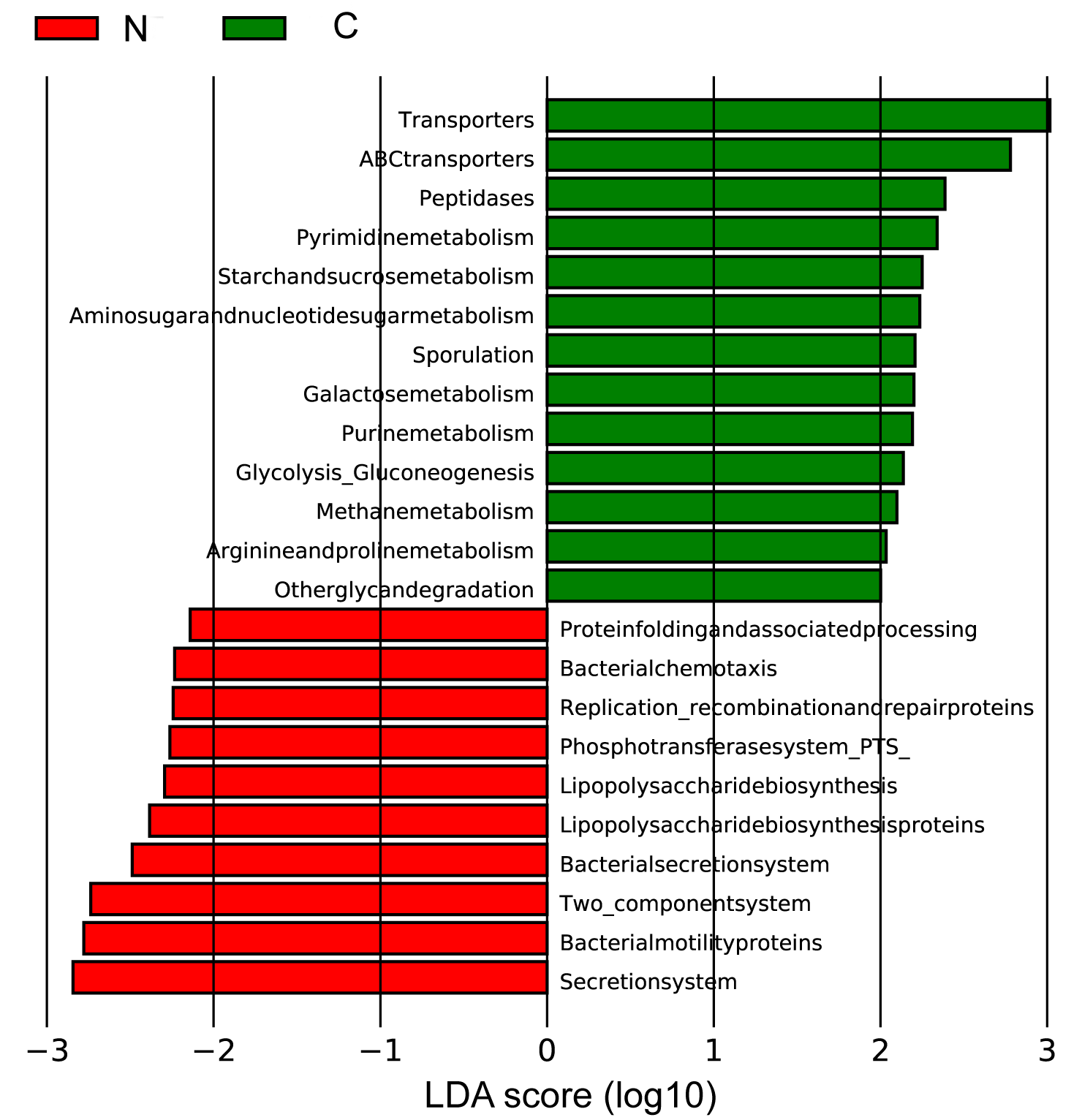

Supplement: FIGURE S3 — Differences in functional compositions between the cancer and non-cancer groups by LEfSe analysis (LDA scores >2.0). Green indicates functions enriched in cancer group and red indicates functions enriched in non-cancer group. C, cancer group; N, non-cancer group. [file Image_3.TIF]
